# Genetic polymorphisms of *Plasmodium falciparum* isolates from Melka-Werer, North East Ethiopia based on the merozoite surface protein-2 (*msp-2*) gene as a molecular marker

**DOI:** 10.1186/s12936-021-03625-1

**Published:** 2021-02-12

**Authors:** Hussein Mohammed, Ashenafi Assefa, Melkie Chernet, Yonas Wuletaw, Robert J. Commons

**Affiliations:** 1 Malaria, Neglected Tropical Diseases Research Team, Bacterial, Parasitic, Zoonotic Diseases Research Directorate, Ethiopian Public Health Institute, Addis Ababa, Ethiopia; 2 Global Health Division, Menzies School of Health Research, Charles Darwin University, Darwin, Australia; 3 Internal Medicine Services, Ballarat Health Services, Ballarat, Australia

**Keywords:** Ethiopia, Genotyping, Polymorphism, Merozoite surface protein 2, Multiplicity of infection

## Abstract

**Background:**

The characterization of parasite populations circulating in malaria endemic areas is necessary to evaluate the success of ongoing interventions and malaria control strategies. This study was designed to investigate the genetic diversity of *Plasmodium falciparum* isolates from the semi-arid area in North East Ethiopia, using the highly polymorphic merozoite surface protein-2 (*msp2*) gene as a molecular marker.

**Methods:**

Dried blood spot isolates were collected from patients with *P. falciparum* infection between September 2014 and January 2015 from Melka-Werer, North East Ethiopia. Parasite DNA was extracted and genotyped using allele-specific nested polymerase chain reactions for *msp2*.

**Results:**

52 isolates were collected with *msp2* identified in 41 (78.8%) isolates. Allele typing of the *msp2* gene detected the 3D7/IC allelic family in 54% and FC27 allelic family in 46%. A total of 14 different *msp2* genotypes were detected including 6 belonging to the 3D7/IC family and 8 to the FC27 family. Forty percent of isolates had multiple genotypes and the overall mean multiplicity of infections (MOI) was 1.2 (95%CI 0.96–1.42). The heterozygosity index was 0.50 for the *msp2* locus. There was no difference in MOI between age groups. A negative correlation between parasite density and multiplicity of infection was found (p = 0.02).

**Conclusion:**

*Plasmodium falciparum* isolates from the semi-arid area of North East Ethiopia are mainly monoclonal with low MOI and limited genetic diversity in the study population.

## Background

In the past decade, malaria morbidity and mortality have decreased significantly worldwide. In 2018, an estimated 228 million cases of malaria occurred worldwide, compared with 251 million cases in 2010 [1]. Ethiopia is one of the African countries where *Plasmodium falciparum* and *Plasmodium vivax* co-exist; with *P. falciparum* accounting for almost 70% of cases [2].

In Ethiopia, malaria remains a major public health problem with an estimated 52% of the population at risk of infection [2, 3]. However, due to improved case management, and the scale-up of long-lasting insecticidal nets (LLINs) and indoor residual spraying (IRS) there has been a significant reduction in the malaria burden, with the malaria programme review in 2020 finding a 67% decline in malaria prevalence from 0.9/100,000 population to 0.3/100,000 population between 2016 and 2020 [4]. The estimated annual parasite index in the Afar region in North East Ethiopia was 52.03 in 2019 compared to 126 in 2013 (Federal Ministry of Health, unpublished data). In most malaria endemic districts, the annual malaria incidence rate is now less than 5% [5, 6]. These successes have prompted the country to move towards malaria elimination strategies [7].

Polymerase chain reaction (PCR)-based genotyping methods are used widely in molecular epidemiological studies to assess allelic diversity and multiplicity of infection [8]. Among the polymorphic genes of *P. falciparum*, merozoite surface protein 1 (*msp1*), *msp2*, and *glurp* markers are used most commonly to differentiate recrudescence of the parasite from new infections in therapeutic efficacy studies [8]. The *msp2* gene is the most conserved and informative single marker for molecular epidemiological studies [9]. MSP2 is a glycoprotein expressed on the surface of merozoites that has been considered as one of the candidates for blood stage malaria vaccines [10]. The *msp2* gene is located on chromosome 2 and is composed of five blocks the most polymorphic of which is the central block 3 [11]. The gene is encoded by highly divergent alleles, grouped into two dimorphic families FC27 and IC/3D7 [12]. Genotyping of *P. falciparum* in malaria endemic areas can be used to determine the genetic diversity of falciparum malaria and multiplicity of infection (MOI), which can be used to infer transmission intensity. For example, studies higher heterozygosity (He) and MOIs have been described in high malaria transmission areas compared with low transmission areas [13, 14].

Several studies have investigated the genetic diversity of *P. falciparum* in endemic regions in Africa, South America and Asia [15–17], including a few studies from Ethiopia [18–21]. Most of these studies were conducted in moderate to high trasmision settings and showed high genetic diversity and MOI. However, no studies have described the diversity of *P. falciparum* from the semi-arid climatic zones of Ethiopia. Given the recently enhanced malaria control interventions in Ethiopia, assessment of the genetic diversity of *P. falciparum* provides an additional understanding of progress towards elimination in the country and a point of comparison for future studies in the region. This study aimed to determine the genetic diversity and multiplicity of *P. falciparum* infection based on *msp2* gene polymorphisms in the semi-arid rural area of North East Ethiopia.

## Methods

### Study site

**Fig. 1:**
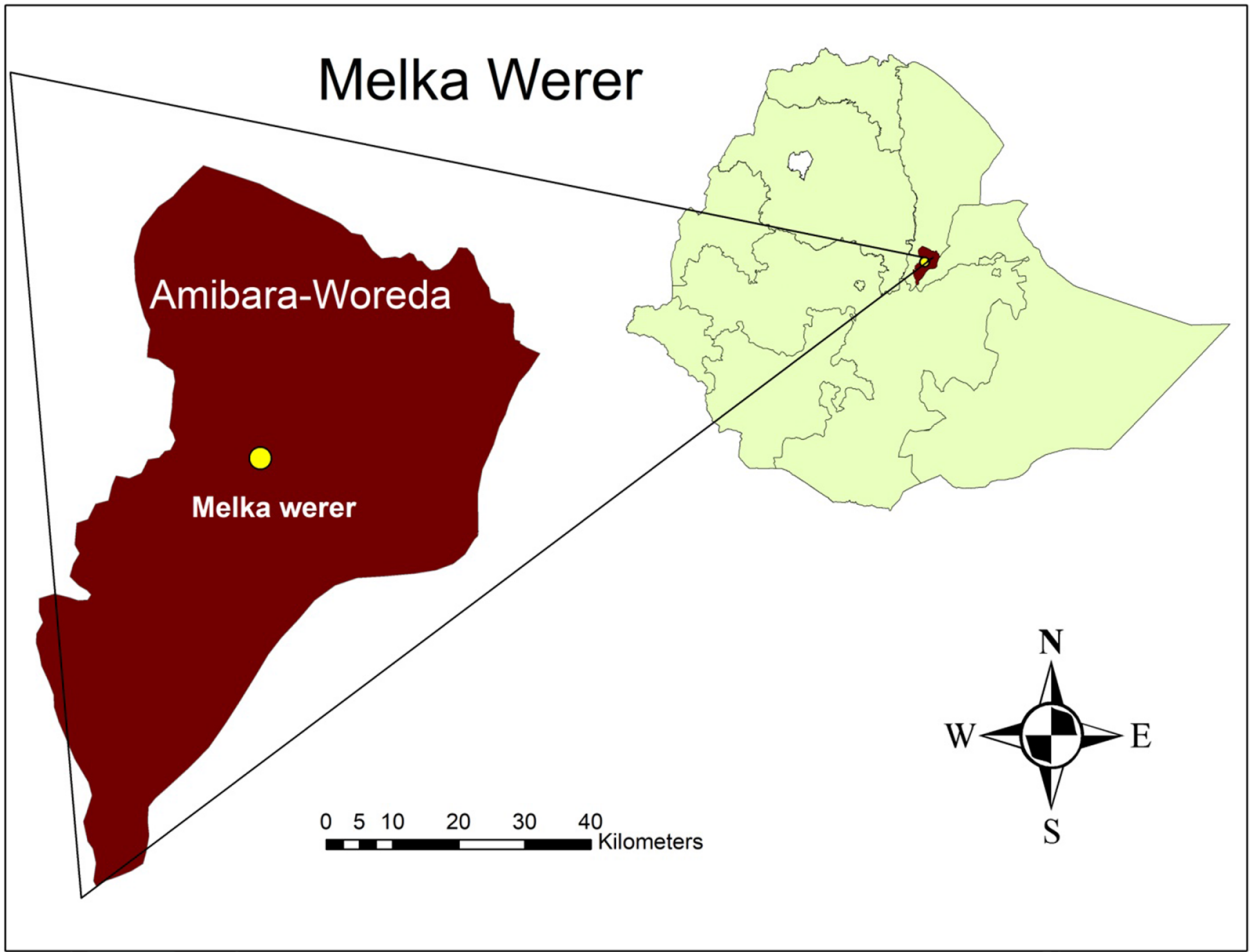
Map of the sample collection area, Meleka-Werere, North East, Ethiopia

The study samples were collected from Melka-Werer rural area in Afar Regional state in North East Ethiopia, a sentinel site for monitoring of anti-malarial therapeutic efficacy (Fig. 1). The study area is located 291 km northeast of Addis Ababa at an altitude of 723 m above sea level. Melka-Werer is one of the *kebeles* of Amibara district, with a catchment population 61,222 inhabitants; the majority living as pastoralists or semi-pastoralists [22]. The semi-arid climatic zone has a long hot summer, and a short mild winter with annual rainfall between 200 and 500 mm. Malaria transmission is markedly seasonal, with a peak during August to December. *Plasmodium falciparum* and *P. vivax* are the two dominant malaria parasites in the region. The local vector responsible for most malaria transmission is *Anopheles arabiensis* with mosquito breeding predominantly occurring adjacent to the Awash River and malaria distribution reflecting this. The prevalence of malaria in this region is declining according to Ethiopian national malaria indicator surveys; with a cross-sectional prevalence of 2.4% in 2007, 0.8% in 2011 and 0.2% in 2015 [6, 23, 24].

**Fig. 2:**
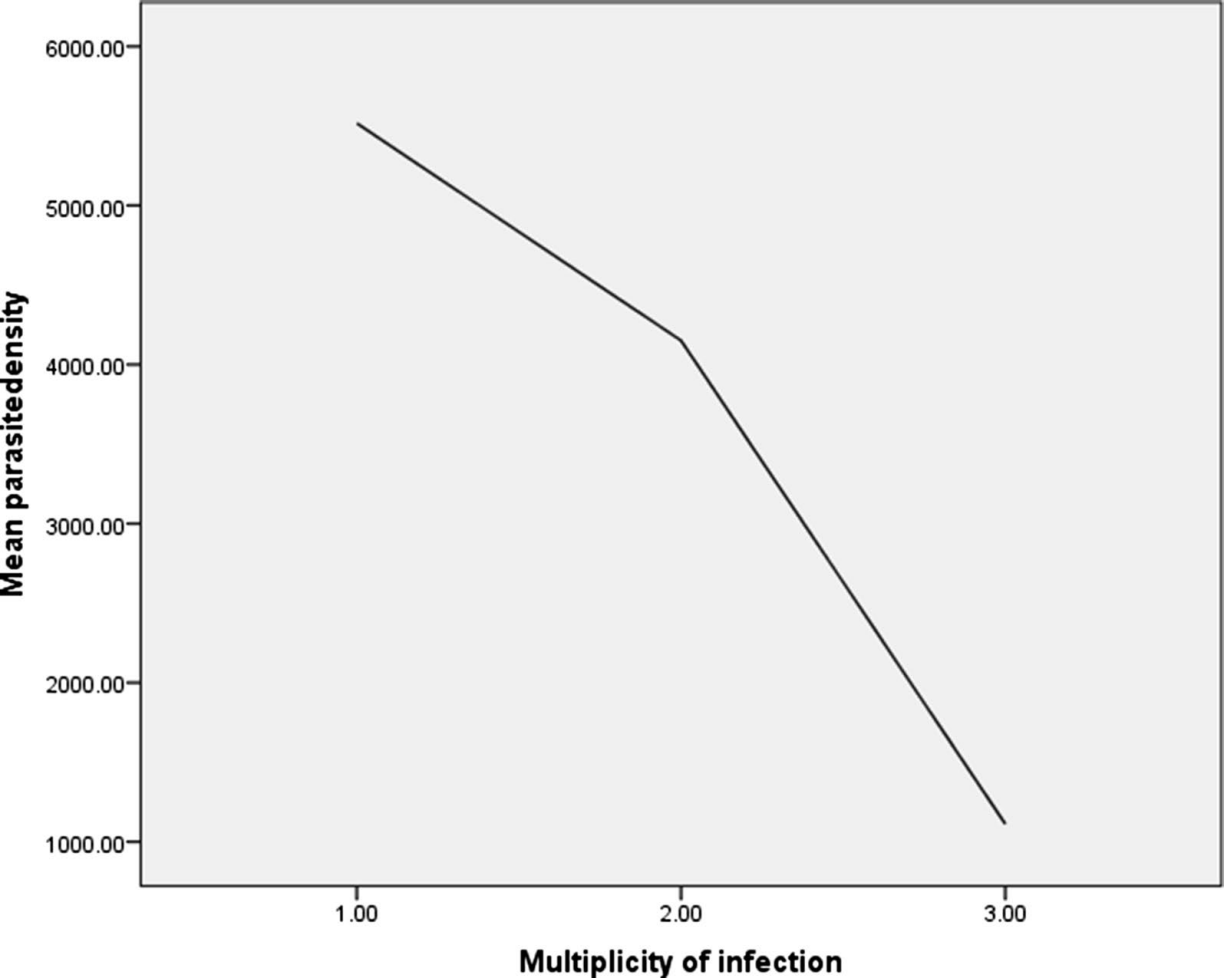
Relationship between mean parasite density and multiplicity of infection of *Plasmodium falciparum* infection

### Study population and sample collection

Dried blood spot samples were collected from children and adults presenting to the Melka-Werer Health Centre with *P. falciparum* malaria and enrolled into a 28-day therapeutic efficacy of artemether-lumefantrine which found 100% adequate clinical and parasitological responses*.* The study was conducted from September 2014 to January 2015. Individuals with an axillary temperature of  ≥ 37.5 °C or history of fever within the previous 24 h, haemoglobin (Hb) level > 5 g/dL, microscopically confirmed *P. falciparum* mono-infection with asexual parasite densities between 1,000 and 100,000 parasites/µL blood and residence within the study area were eligible for enrolment in the study [25]. Thick and thin blood films were stained with 3% Giemsa for 45 min and slides were read by two health centre laboratory technicians. In case of discordant results, a third WHO-certified microscopist resolved the discrepancy. Using the thick blood film, asexual parasitaemia was counted against 200 leucocytes and expressed as the number of asexual parasites/μL of blood, assuming a leucocyte count of 8,000/μL of blood [26]. Haemoglobin was measured from finger prick blood samples using a portable spectrophotometer (HemoCue Ängelholm Sweden). Before treatment, approximately 50 µL of blood from each patient was spotted onto Whatman 903® filter paper (Schleicher & Schuell BioScience), air-dried and stored in labelled zip lock bags with desiccant before being transported and stored at − 20 °C from 2015 to 2020 in the Malaria Research Laboratory at the Ethiopian Public Health Institute (Fig. 2).

### Parasite DNA extraction and molecular genotyping

DNA was extracted from the dried blood spots using the Chelex-Saponin method [27] and allelic family-specific analyses of *msp2* block 3 were carried out as previously described [20]. The allele- specific positive control 3D7 and DNA free negative controls were included in each set of reactions [16]. The nested PCR products were separated by electrophoresis on 2% agarose gel in 1X TBE (Tris borate EDTA) buffer stained with 0.5% (v/v) ethidium bromide at 80 V for 30 min. After running the gel, it was placed in a gel documentation system (Cleaver Scientific UV Transilluminator, UK) that was connected to a desktop computer to visualize the bands under ultraviolet transillumination. The size of DNA fragments was estimated visually based on their mobility related to a 100 bp DNA ladder marker (Boehringer Mannheim Marker VI). Alleles in each family were considered the same if fragment size was within a 20 bp interval [28]. The MOI was calculated by dividing the total number of distinct *msp2* fragments observed by the number of positive samples. Isolates with a single genotype were considered monoclonal infections and those with more than one genotype as polyclonal infections. The frequency of monoclonal infections was the number of patients with one parasite genotype divided by the total infected population. Heterozygosity index was calculated using the following formula He = n/(n−1) (1- ΣPi^2^), where n is the number of isolates sampled and Pi is the allele frequency [29].

### Statistical analysis

Data were recorded in Excel and analysed with SPSS version 20 (SPSS Inc., Chicago, IL, USA). The frequency of *msp2* allelic families was calculated as a proportion of all detected alleles in the isolates. Spearman’s rank correlation coefficient was calculated to assess the association between MOI and mean parasite density and age. The non-parametric Mann–Whitney U test was used to compare the association between MOI and previous exposure to malaria. A *P* value < 0.05 was considered statistically significant.

## Results

### Study population

A total of 72 patients were confirmed to have malaria. Of these, 52 (72%) patients were confirmed to have *P. falciparum* malaria and were genotyped for the *msp2* alleles. An equal proportion of males (26, 50%) and females (26, 50%) were enrolled. The patients’ ages ranged from two to 60 years (mean 21.3 ± 14.4). The geometric mean parasitaemia was 3,661 (95% CI 2,768–4,877) ranging from 1,000 to 83,200 parasites/µL (Table 1). The mean axillary temperature was 38.1 °C (95% CI 37.8–38.4). 90.4% of the study participants could not recall previous exposure to malaria. The geometric mean parasite density of individuals with previous exposure to malaria attack was higher 10,854 parasites/µL compared with 3,226 parasites/µL in those without prior exposure.

**Table 1:** Demographic and parasitological characteristics of the study population from Melka-Werer, North East Ethiopia

Characteristic	Values
Sex ratio (Male/Female)	1 (26/26)
Mean age ± SD (years)	21.3 ± 14.4
Age range (years)	2–60
Age group	
Children (< 10 years)	13 (25.0%)
Children (10–20 years)	14 (26.9%)
Adults (> 20 years)	22 (48.1%)
Geometric mean parasitemia (95% CI) (p/µL)	3660.8 (2768.3–4877.2)
Parasite density range (p/µL)	1000–83,200
Mean axillary temperature (95% CI) (^o^C)	38.1 (37.8–38.4)
Mean haemoglobin ± SD (g/dL)	11.9 ± 1.8

*SD* standard deviation, *p/µl* parasite per microliter

### Allele frequencies

All samples positive for *P. falciparum* were genotyped for *msp2* Block-3 by nested PCR. *msp2* was identified in 41 (78.8%) samples. A total of fourteen different alleles were identified in *msp2* genotyping. Six alleles of 3D7/IC (280–500 bp) and 8 alleles of FC27 (180–450 bp) were detected. The distribution of the corresponding band size is presented in Additional file 1: Figure S1. The proportion of isolates with only 3D7/IC and FC27 alleles was 34.1% (14/41) and 22.0% (9/41), respectively. Both *msp2* allelic families were identified in 43.9% (18/41) of the isolates (Table 2). 40.4% (21/52) of the isolates contained multiple *msp2* alleles and the overall mean MOI was 1.2 (95% CI 1.00–1.40). The heterozygosity index for the *msp2* locus was 0.5.

**Table 2:** Distribution of *msp2* allelic families of *P. falciparum* isolates from Melka-Werer, North East Ethiopia

Allelic family	n (%)	Fragment size (bp)	No. of alleles	Mean MOI
*msp2*	41 (100)			1.2 (95% CI 1.00–1.40)
FC27	9 (22)	180–450 bp	8	1.04 (95% CI 0.96–1.11)
3D7/IC	14 (34)	280–500 bp	6	1.06 (95% CI 0.97–1.15)
FC27 + 3D7/IC	18 (44)			
Total	41		14	

*bp* base pair, *CI* Confidence Interval, *n* number of samples, *MOI *multiplicity of infection

### Relationship between MOI, parasite density and age

A negative correlation was observed between parasite density and MOI (Spearman rank coefficient = − 0.33 *P* = 0.022). Age was not correlated with the MOI (Spearman rank coefficient = 0.080; *P* = 0.51). The highest MOI was in the 10–18-year-old age group as shown in Table 3. There was no association between the MOI of individuals with previous exposure to malaria attack compared to those without previous exposure (p = 0.935).

**Table 3:** Mean parasitemia and multiplicity of infection of *Plasmodium falciparum msp2* gene stratified by age group (n = 52)

Age (years)	n (%)	Parasite density (parasite/µL)	Mean MOI
< 10	13 (25.0)	12,382	1.00
10–18	14 (26.9)	4754	1.50
> 18	25 (48.1)	4986	1.12

*n*  number of malaria cases, *MOI*  multiplicity of infection

## Discussion

This study was conducted to assess the current *P. falciparum* genotypic structure in the semi-arid area in North East Ethiopia, using the highly polymorphic (block 3) region of the *msp2* gene as a molecular marker. The *msp2* marker is recommended for genotyping *P. falciparum* parasite populations compared with *msp-1* and *glurp* [30]. *Plasmodium falciparum* isolates from this region were mainly monoclonal with a low MOI and limited genetic diversity. These findings are important for ongoing evaluation of the effect of malaria control strategies, as Ethiopia moves towards malaria elimination.

The 3D7/IC allelic family of *msp2* was more prevalent than the FC27 allelic family. This is in agreement with previous reports from Burkina Faso [31], South West Ethiopia [22] and Sudan [32]. However, this finding differs to results from North West Ethiopia [20], and Central Sudan [33], where FC27 was the more prevalent allelic family. These differences could relate to the semi-arid geographic setting and low transmission intensity compared to the hot and humid climate in North West Ethiopia.

Limited genetic diversity of *P. falciparum* was observed in this study. Similar results have been reported in other areas with low *P. falciparum* transmission [34] and in regions with declining transmission related to malaria control efforts [35]. In contrast, a high level of genetic diversity was reported in high endemicity settings in Cameroon [15] and Burkina Faso [31].

The current study found that the *P. falciparum* parasite population in Melka-Werer exhibited a low heterozygosity (He = 0.5), consistent with that reported in Mubuga, Rwanda (He = 0.49) [36]. In areas with declining local transmission, it is expected that lower parasite diversity (heterozygosity) will be present [37]. Declining diversity and transmission have been associated with improved malaria control interventions [38, 39].

The overall mean MOI reported in this study was low (MOI = 1.2). This is in agreement to previous studies where low malaria transmission settings are commonly associated with lower MOIs [40, 41], and is consistent with reports from semi-desert settings in neighbouring Sudan and Djibouti [32, 38]. The low MOI contrasted with a finding from a higher endemic setting in Humera, Ethiopia [19]. The low MOI observed in this study may reflect most positive samples being from adult patients, with previous reports finding a reduction in MOI in adults compared with children [43].

The majority of participants in the current study were older than 10 years, similar to results from an area with a lower intensity of malaria transmission [31] but contrasting to reports from high transmission settings [44]. It is also possible that the age-related malaria risk may have been influenced by implementation of effective malaria control interventions, such as the widespread distribution of long-lasting insecticidal nets (LLINs) and indoor residual spray (IRS), and sustained treatment of malaria patients with artemisinin-based combination therapy (ACT). This is supported by the 2015 malaria indicator survey, which found that the Afar region had the highest percentage of use of LLINs compared to other regions of the country [6].

Age is considered an important factor in the acquisition of immunity against *P. falciparum* and may have also an effect on MOI [45], although, the influence of age on the MOI is highly affected by malaria transmission intensity [46]. Previous studies have shown an association between age and MOI in areas with intense perennial malaria transmission or hypo-meso-endemic malaria transmission [47, 48]. However, the current study found no association between age and MOI. Similar findings have been reported in other countries [49, 50].

A higher geometric mean parasite density in individuals with previous exposure to malaria attack was found. In this low endemicity setting, a lower proportion of individuals will have likely had prior immunity, meaning that infected patients will be more likely to become symptomatic at a lower parasitaemia than in high endemicity settings.

It was difficult to correlate transmission levels with genetic diversity and MOI in the current study due to a lack of entomologic inoculation rate (EIR) data from the study area. However, the genetic diversity and the MOI reported in the present study supported a low average microscopy positivity rate (8.5%) (Melka-Werer rural town health office data, 2015, unpublished). A limitation of this study was the small sample size, in part due to the nomadic nature of the local communities. Furthermore, due to resource restrictions, lower discriminatory power agarose gel electrophoresis compared to capillary electrophoresis was used [51]. Further, the limited allelic frequency and genetic diversity observed may have been due to the detection limit of the PCR technique used in the study. Allelic fragment length intervals of less than 20 base pairs may not be clearly distinguished on agarose gel and may lead to misclassification of the genotype. Allele differentiation could be improved by using more discriminatory techniques in future studies, such as DNA sequencing or SNPs.

## Conclusion

This study found limited genetic diversity of *P. falciparum* isolates from the semi-arid area of North East Ethiopia, with most infections monoclonal. This correlates with the low prevalence of infection in this region. There is a need for further studies in similar low transmission settings with larger sample sizes using capillary electrophoresis to further investigate the dynamics of falciparum malaria diversity in such regions of Ethiopia.

## Supplementary Information

**Additional file 1: Fig. S1**.

## Data Availability

The dataset supporting the conclusion of this article of this article is included within the article and the additional files.

## Abbreviations

bp: Base pair
msp2: Merozoite surface protein 2
MOI: Multiplicity of infection
PCR: Polymerase chain reaction
