# Supplementary figures and images for: Genetic polymorphisms of Plasmodium falciparum isolates from Melka-Werer, North East Ethiopia based on the merozoite surface protein-2 (msp-2) gene as a molecular marker
Source: Malar J. 2021 Feb 12;20:85. doi: 10.1186/s12936-021-03625-1 (PMC7881608; doi:10.1186/s12936-021-03625-1)

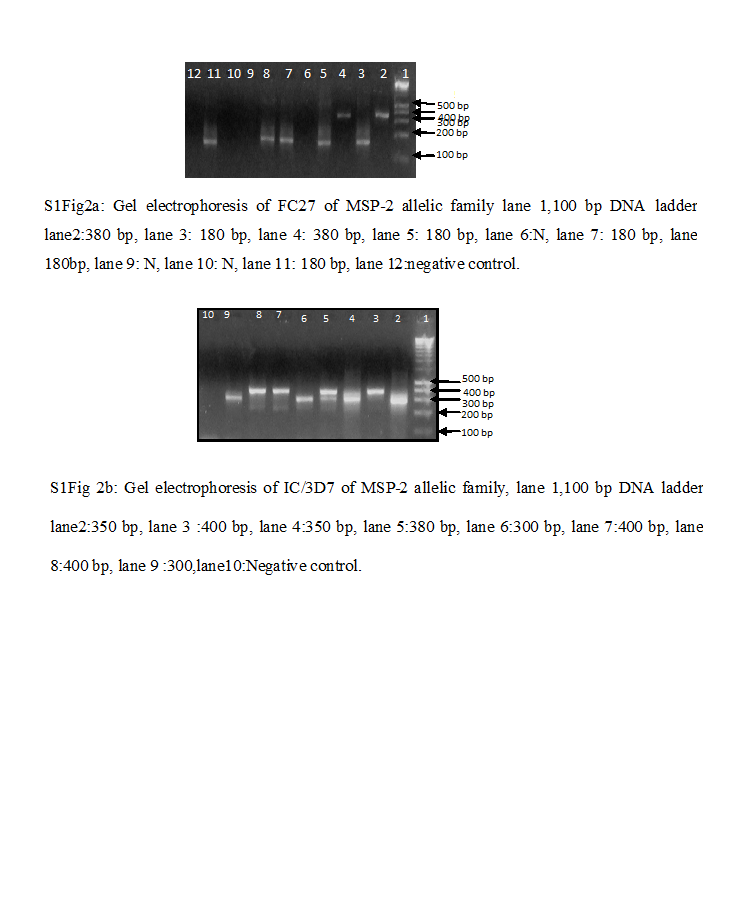

Supplement: Supplementary file 1 — Additional file 1: Fig. S1. [file 12936_2021_3625_MOESM1_ESM.png]
